# Role of adenosine Stress CMR before Chronic Total Occlusion reopening

**DOI:** 10.1186/1532-429X-18-S1-P80

**Published:** 2016-01-27

**Authors:** Lorenzo Monti, Gabriella Di Giovine, Claudia Scardino, Barbara Nardi, Luca Balzarini, Gabriele Gasparini

**Affiliations:** 1 grid.417728.f0000000417568807Radiology, Humanitas Research Hospital, Rozzano (MI), Italy; 2 grid.417728.f0000000417568807Cardiology department, Istituto Clinico Humanitas, Rozzano, Italy; 3 Cardiology, Ospedale di Novara, Novara, Italy; 4 grid.414818.00000000417578749Cardiology, IRCCS Ospedale Maggiore Policlinico di Milano, Milano, Italy

## Background

Conflicting data exists on the usefulness of a perfusion stress test prior to reopening a chronically occluded coronary artery (CTO). We sought to test whether a stress MR may improve patient selection for Chronic Total Occlusion (CTO) reopening over a conventional LGE study.

## Methods

70 CTO patients without any other relevant coronary artery stenosis (or already treated if present at baseline angiography), underwent an adenosine stress CMR before a reopening attempt. ECG and echocardiographic (echo) data were available for all. Patients were considered suitable for the reopening procedure in case of myocardial ischemia and / or viable myocardium subtended to the CTO.

Presence of a previous myocardial infarction (MI) was defined at ECG: Q waves > 40 msec; at echo: akinetic areas with reduced wall thickness; at stress MR: presence of ischemic Late gadolinium Enhancement.

Presence of myocardial ischemia was defined as a subendocardial perfusion defect lasting for at least 4 heart beats during adenosine infusion in a segment without LGE.

## Results

ISCHEMIA: adenosine stress MR showed a perfusion abnormality in 100% of CTO territory. Inducible ischemia was observed in 71% of cases, with a perfect concordance with CTO occlusion.

PREVIOUS MI: using CMR as the gold standard for the diagnosis, the prevalence was globally high: 69% in our series. Both ECG and echo significantly underestimate this data (36% and 53% respectively). Presence of LGE in different sites from the CTO territory was often observed: in fact, only 58% of patients with LGE had it exactly in the CTO territory. At echo, 82% of pts with wall motion abnormality (WMA) had it described in CTO segments: this can be explained with a portion of patients with viable, non-ischemic myocardium. The true prevalence of LGE in CTO segments was 40% for the whole population, but only 13% (n = 9) of patients showed a mean LGE transmurality >50%, contraindicating a reopening attempt. These patients were not identified by echo that identified 30 patients with akinetic areas subtended to a CTO.

## Conclusions

Perfusion stress MR did not show any additional diagnostic value in our population of patients with CTO and known coronary anatomy, since a perfusion abnormality was observed in all patients.

LGE analysis showed a 13% prevalence of transmural MI in CTO territories: this important information can't be obtained with a baseline diagnostic approach and should encourage the use of LGE MR before a CTO reopening.

**Table 1:** Previous MI in CTO patients

	ECG (Q waves)	ECHO (akinesia + wall < 6 mm)	Ischemic LGE pattern
Global prevalence of diagnostic criterion	36% (n = 25)	53% (n = 37)	69% (n = 48)
Correct site of a positive criterion	80% (n = 20)	81% (n = 30)	58% (n = 28)
Previous MI in the CTO territory	29% (n = 20)	43%(n = 30)	40% (n = 28)

